# The role of genetic testing in the diagnostic workflow of pediatric patients with kidney diseases: the experience of a single institution

**DOI:** 10.1186/s40246-023-00456-w

**Published:** 2023-02-13

**Authors:** Tiziana Vaisitti, Valeria Bracciamà, Angelo Corso Faini, Giulia Margherita Brach Del Prever, Martina Callegari, Silvia Kalantari, Fiorenza Mioli, Carmelo Maria Romeo, Maria Luca, Roberta Camilla, Francesca Mattozzi, Bruno Gianoglio, Licia Peruzzi, Antonio Amoroso, Silvia Deaglio

**Affiliations:** 1 Immunogenetics and Transplant Biology Service, University Hospital “Città della Salute e della Scienza di Torino”, Via Santena 19, 10126 Turin, Italy; 2 Department of Medical Sciences, University of Turin, Via Nizza 52, 10126 Turin, Italy; 3 Pediatric Nephrology Dialysis and Transplantation, University Hospital “Città della Salute e della Scienza di Torino”, Turin, Italy

**Keywords:** Clinical exome sequencing, Next-generation sequencing, Kidney diseases, Genetic testing, Pediatric cohort

## Abstract

**Purpose:**

Inherited kidney diseases are among the leading causes of kidney failure in children, resulting in increased mortality, high healthcare costs and need for organ transplantation. Next-generation sequencing technologies can help in the diagnosis of rare monogenic conditions, allowing for optimized medical management and therapeutic choices.

**Methods:**

Clinical exome sequencing (CES) was performed on a cohort of 191 pediatric patients from a single institution, followed by Sanger sequencing to confirm identified variants and for family segregation studies.

**Results:**

All patients had a clinical diagnosis of kidney disease: the main disease categories were glomerular diseases (32.5%), ciliopathies (20.4%), CAKUT (17.8%), nephrolithiasis (11.5%) and tubular disease (10.5%). 7.3% of patients presented with other conditions. A conclusive genetic test, based on CES and Sanger validation, was obtained in 37.1% of patients. The highest detection rate was obtained for ciliopathies (74.4%), followed by nephrolithiasis (45.5%), tubular diseases (45%), while most glomerular diseases and CAKUT remained undiagnosed.

**Conclusions:**

Results indicate that genetic testing consistently used in the diagnostic workflow of children with chronic kidney disease can (i) confirm clinical diagnosis, (ii) provide early diagnosis in the case of inherited conditions, (iii) find the genetic cause of previously unrecognized diseases and (iv) tailor transplantation programs.

**Supplementary Information:**

The online version contains supplementary material available at 10.1186/s40246-023-00456-w.

## Introduction

Pediatric nephropathies comprise widely different disease entities in terms of clinical presentation, evolution, and therapeutic options [1–4]. Approximately 30% of children with chronic kidney disease (CKD) suffer from a monogenic condition, a percentage increasing when considering children with end-stage renal disease (ESRD) [5, 6]. Many of these children remain undiagnosed at the time of transplantation [4, 6, 7]. Reaching a diagnosis for these patients not only implies the end of a diagnostic odyssey, but presents several advantages for prognosis, management, and treatment.

Pioneering studies have consistently shown that the implementation of next-generation sequencing (NGS) techniques has significantly improved the diagnostic yield in patients with inherited kidney diseases (IKD) [8]. More recently, the widespread use of NGS has made available genetic diagnosis in a reasonable time and at affordable costs, raising the question of whether and when it should be integrated in the routine diagnostic workflow [5].

Genetic diagnosis in children is of utmost importance for different aspects. The first is that it may be relevant in the clinical approach to the disease, the typical example being that of nephrotic syndromes where the identification of structural variants in podocyte-related genes argues against immunosuppressive therapies that would otherwise be routinely used over a period of several months [9]. The second is that it may be highly relevant for the child’s family and for the identification of other members who are carrying the variant possibly transmissible to future generations. Once a pathogenic variant is identified in a proband, cascade testing of family members and genetic counselling in variant-carriers represent a standard practice in clinical genetics [5]. The third is that knowing the pathogenic variant is essential in the transplantation context where the donor may be a relative. Indeed, it is critical to rule out the presence of the same variant(s) in the organ donor, as well as it is critical to identify all family members potentially in need of a transplant. The fourth is that some diseases present a high risk of relapse after the organ transplantation, such as the focal segmental glomerulosclerosis [10] or their outcome may be improved by a more tailored choice of the transplant to be performed, such as in the case of primary hyperoxaluria where a combined kidney-liver transplant may result in a better outcome [11]. Finally, having a clear disease diagnosis may be useful for the patient to take part in clinical trials and to benefit from novel treatment options [12, 13].

At the end of 2018, in a collaboration between pediatric nephrologists and geneticists, we started performing genetic tests for monogenic conditions potentially leading to ESRD and hence transplantation. Our hospital is the biggest in Northwest Italy, draining from an area of about 5 million people. We selected a “one size fits all” kind of analysis, with sequencing of the clinical exome, i.e., approximately 6700 genes that are associated to monogenic conditions, focusing analysis on gene panels tailored on the clinical suspicion and therefore limiting incidental findings and reducing time for sequence analysis.

Overall, by applying this pipeline, we obtained a diagnostic yield in line with published data, with some heterogeneity among the different clinical suspicion, as expected. The results obtained confirm the relevance of including routine genetic testing and counselling in the diagnostic workflow of pediatric patients affected by nephropathies. Indeed, the identification of causative variants is critical for their clinical management, and potentially for optimal live-donor selection.

## Materials and methods

### Patients’ recruitment

The study was based on a diagnostic cohort of 191 consecutive pediatric patients (age at recruitment < 18 years old), recruited by the Pediatric Nephrology, Dialysis and Transplantation Units at the Regina Margherita Children’s Hospital and referred to the Immunogenetics and Transplant Biology Service for genetic analysis. All patients included in the study provided a written informed consent signed by both parents, whenever possible.

### Sample preparation, sequencing, and bioinformatics analyses

Nucleic acid extraction from peripheral blood, analysis of DNA quality, library preparation and sequencing were performed as previously reported [14]. Raw data obtained from sequencing were converted in FASTQ files and then aligned with Enrichment 3.1.0 or DRAGEN Enrichment tools (Illumina) and mapped on TruSightOne Expanded v2.0 manifest using Homo Sapiens UCSC GRCh37 genome as reference to obtain single nucleotide variants, copy number variants (CNV) and structural variants vcf files. For copy number identification, a baseline made of sequencing data from 5 different patients, all negative for CNV (as per array comparative genomic hybridization data) was used. This approach allows to detect CNV even in sexual chromosomes since the reference group was made both of female and male individuals and gender of the subject to be analyzed was always specified during the alignment phase. Variants calling and prioritization was made following defined criteria. Reads alignment and exons coverage of gene of interest were checked and displayed by Integrative Genomics Viewer-IGV, freely available from the UC San Diego—University of California and the Broad Institute of MIT and Harvard University—Boston (https://software.broadinstitute.org/software/igv/). Variants to be included in the final genetic report were classified according to the American College of Medical Genetics and Genomics (ACMG) criteria.

### Generation of in silico gene-disease list

Genes to be considered for variants identification and prioritization were defined based on the clinical suspicions. In silico gene lists were generated matching (i) data from different databases, correlating genotype to phenotype (OMIM, PanelApp England, ClinGen, Malacards), and (ii) data from literature. The available gene lists are updated once a year based on novel evidence of gene-disease association.

### Sanger sequencing and multiplex ligation-dependent probe amplification (MLPA)

Sanger sequencing and/or MLPA analyses were performed to validate variants identified by NGS and for family segregation studies. Briefly, DNA was extracted starting from a second independent aliquot of the proband peripheral blood and from the parents. The DNA regions of interest were amplified by PCR using specific experimental conditions. The purity and specificity of the amplified regions were checked by 0.8 or 1.5% agarose gel. Amplified PCR products were then Sanger sequenced using the same primers. For *PKD1* variants, validation was performed using a long-range PCR followed by a nested PCR to avoid any inference from the pseudogenes. Electropherograms were then analyzed using the Chromas software version 2.6, freely available at www.technelysium.com.au.

## Results

### Definition of criteria for the return of genetic analysis results

We previously reported on the design and set-up of a “kidney” gene panel that comprises > 400 genes all involved in different forms of kidney diseases [14]. For this study, we implemented analysis with subpanels focused on the specific clinical category of suspicion (e.g., CAKUT, glomerulopathy, tubulopathy, etc.) and prioritized a specific group of genes for analysis. This approach limited the number of analyzed genes, simplifying analyses, and reducing the number of incidental findings. Only when, at the end of analytical flow with relevant panel(s), the genetic result was negative and (i) the clinical phenotype not clearly indicated or (ii) overlapping different disease categories, genetic analysis was extended to the so-called “kidney full-list or kidneyome”, a super-panel comprising all the genes included in the subpanels.

**Fig. 1:**
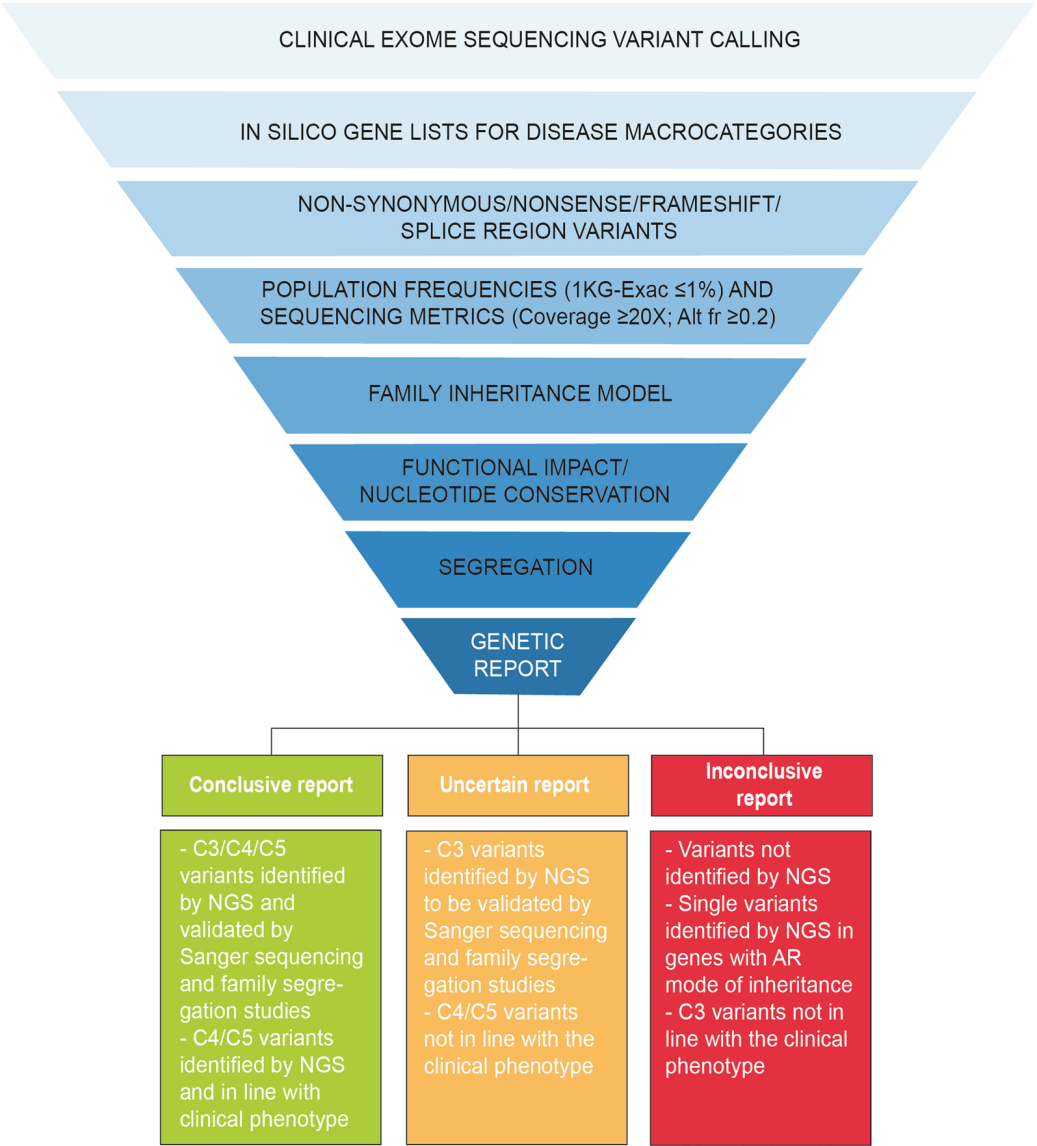
Genetic data analysis pipeline and criteria for variant inclusion. Schematic representation of the analytical pipeline adopted for variant identification and prioritization, including all the filtering-in and filtering-out criteria. The resulting variants were included in the final genetic report. Whenever possible, variant(s) validation and family segregation studies were performed. Genetic reports were classified as conclusive, uncertain, or inconclusive based on the indicated criteria. Based on this classification the diagnostic rate of our next-generation sequencing (NGS) workflow was calculated. 1 KG: 1000 genomes database; Alt fr: altered frequency; C3: variant of unknown significance; C4: likely pathogenic variant; C5: pathogenic variant; AR: autosomal recessive

As a first step, we defined a set of criteria for interpretation of NGS results. After performing clinical exome sequencing (CES) and data alignment, a pipeline of analysis was determined to filter-in the relevant variants. Specifically, based on the clinical suspicion, identified variants were filtered based on in silico gene lists, specific for the different disease macro-categories. Synonymous variants not impacting on the splicing mechanism or intronic variants not mapping within the splicing region were excluded, keeping in consideration only the non-synonymous, nonsense, frameshift, and splicing-affecting variants. Then, only rare variants (frequency less than 1% in the population) and variants with an allele frequency in the patient of at least 0.2 and a coverage of at least 20 reads were included. The remaining variants were annotated and further curated based on (i) mode of inheritance, (ii) nucleotide conservation, (iii) protein impact, exploiting different databases to check the scores, and (iv) literature, if any. At this point, filtered-in variants were listed in a so-called “technical report”. The technical report was interpreted by a medical geneticist to produce the final genetic report for the patient and his/her family. During the genetic consult, family segregation studies were proposed to (i) confirm the variants in the proband, and (ii) to include/exclude non-causative variants based on their segregation in the family (Fig. 1).

By adopting these criteria, we were able to define three different categories of genetic report. First, a “conclusive report” that included pathogenic (C5) and likely pathogenic (C4) variants. Reports of variants of unknown significance-VUS (C3) were considered conclusive only if fully compatible with the clinical picture and if family segregation studies confirmed their possible role. Second, an “uncertain report” that included C3 variants identified by CES that are not yet or could not be validated in the context of family segregation studies or C4/C5 variants that were not fully in line with the clinical phenotype. Third, an “inconclusive report” that included (i) a negative CES analysis, meaning that no variants were identified by NGS; (ii) single variants in recessive genes; and (iii) C3 variants not in line with the clinical phenotype, identified when the analysis was extended to all kidney-disease-related genes (Fig. 1).

### Main features of the recruited cohort

This study describes a cohort of 191 pediatric patients (0–18 years of age) who were consecutively referred by the Pediatric Nephrology Unit for genetic analysis from November 2018 to May 2022, with an average of 50 new patients enrolled each year. Criteria for genetic testing were (i) nephropathy associated to positive family history for kidney disease or (ii) clinical suspicion of a monogenic condition or (iii) need to rule out a monogenic condition (as in the case of nephrotic syndromes, where distinguishing monogenic versus non-monogenic diseases is clinically meaningful for prognosis and treatment).

**Fig. 2:**
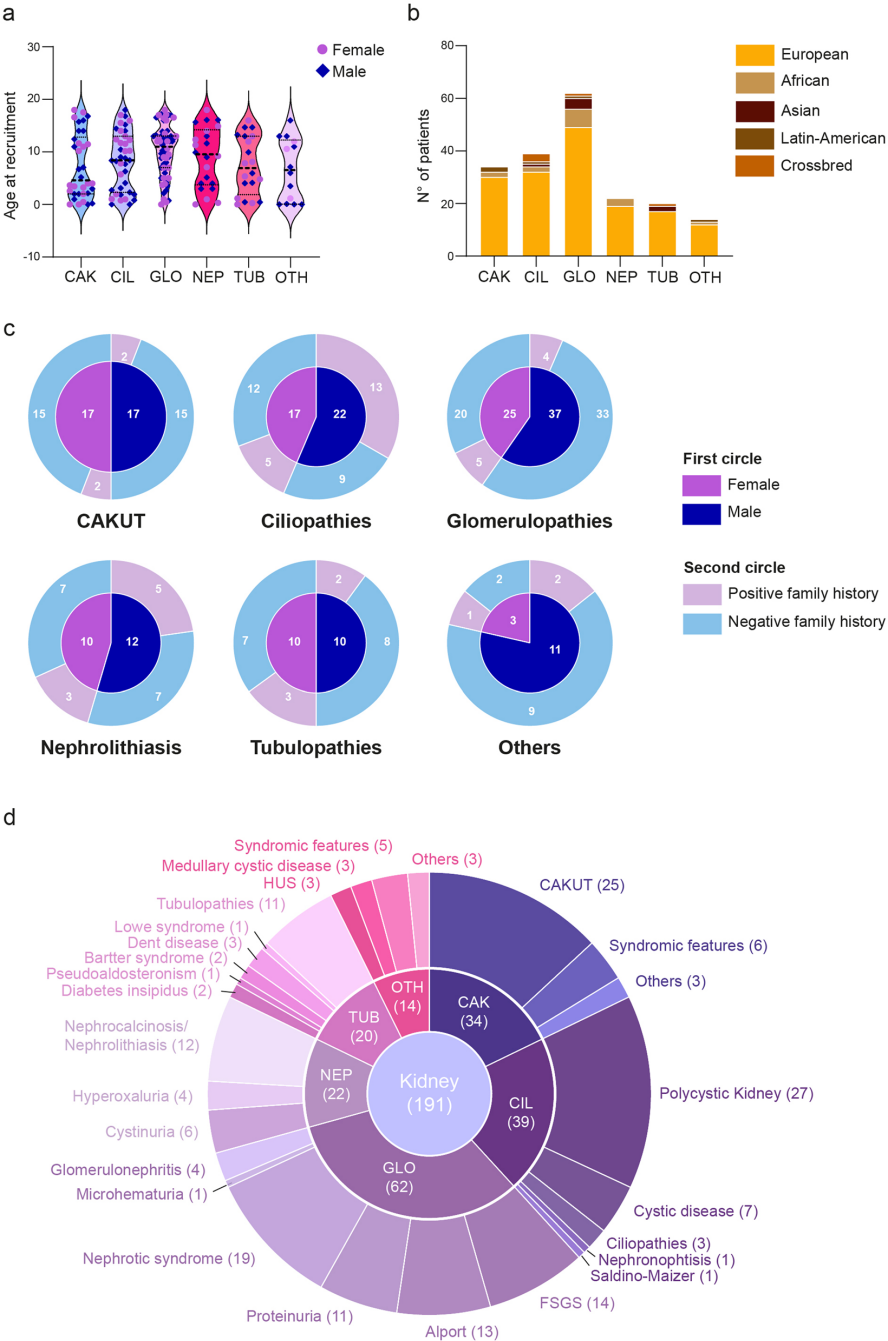
Main features of the pediatric cohort of the study. Distribution of patients according to clinical suspicion and age at recruitment. Data are shown as a violin plot: purple dots represent female subjects while blue diamonds represent male patients. Median and quartiles are shown (**a**). Distribution of patients according to clinical suspicion and ethnicity. The majority of patients are of Caucasian origin, with only a minority of patients being of African origin, Asian, Latin-American or of mixed origin (**b**). Distribution of patients divided on the basis of the main disease macro-categories, according to gender (inner circle) and family history (outer circle) (**c**). Detailed description of the primary disease affecting the pediatric cohort analyzed. Inner circle represents the main macro-categories while the outer circle refers to primary diseases within each macro-category. Numbers in brackets indicate the number of patients (**d**) CAK: congenital abnormalities of kidney and urinary tract (CAKUT); CIL: ciliopathies; GLO: glomerulopathies; NEP: nephrolithiasis; TUB: tubulopathies; OTH: others; HUS: Hemolytic uremic syndrome; FSGS: focal segmental glomerulosclerosis.

The cohort was divided on the basis of the clinical suspicion, considering 6 different disease macro-categories: congenital abnormalities of kidney and urinary tract (CAKUT; *n* = 34), ciliopathies (*n* = 39), glomerulopathies (*n* = 62), nephrolithiasis (*n* = 22), tubulopathies (*n* = 20) and other diseases that included also syndromic phenotypes (*n* = 14). Except for CAKUT and tubulopathies that showed an equal distribution between females and males, in all the other categories there was a prevalence of male subjects (Fig. 2a). When looking at the age of recruitment, no significant different distribution was highlighted among the different groups, with mean age ranging from 6.7 to 10 years old in “Other diseases” and glomerulopathies, respectively. However, when looking at the median age, CAKUT diseases showed the lowest value (4.6 years old), in keeping with a congenital phenotype. From the ethnicity point of view, independently of the disease macro-category considered, most patients were European, followed by African, with only few patients being Asian, Latin-American or crossbred (Fig. 2b). Among the cohort, only 8 patients had consanguineous parents.

Finally, when taking into account family history for kidney diseases considering male and female subjects separately, heterogeneity in distribution between positive and negative cases was evident considering the different disease categories. Overall, most of the recruited cohort and independently of the gender was not characterized by a positive family history, as shown for CAKUT, glomerulopathies, tubulopathies and other kidney disorders. However, in ciliopathies and nephrolithiasis, a significant proportion of cases presented with a positive family history with a different distribution between females and males (13 cases out of 22 for ciliopathies, and 5 out of 12 for nephrolithiasis; Fig. 2c).

From the clinical standpoint, the cohort was quite heterogenous with different primary diseases included (Fig. 2d). Among them, the most recurrent clinical suspicions were polycystic kidney disease (*n* = 27), CAKUT (*n* = 25), nephrotic syndrome (*n* = 19), focal segmental glomerulosclerosis (FSGS; *n* = 14) and Alport syndrome (*n* = 13; Fig. 2d).

### CES and family segregation studies allowed the identification of causative variants in a significant proportion of patients

**Fig. 3:**
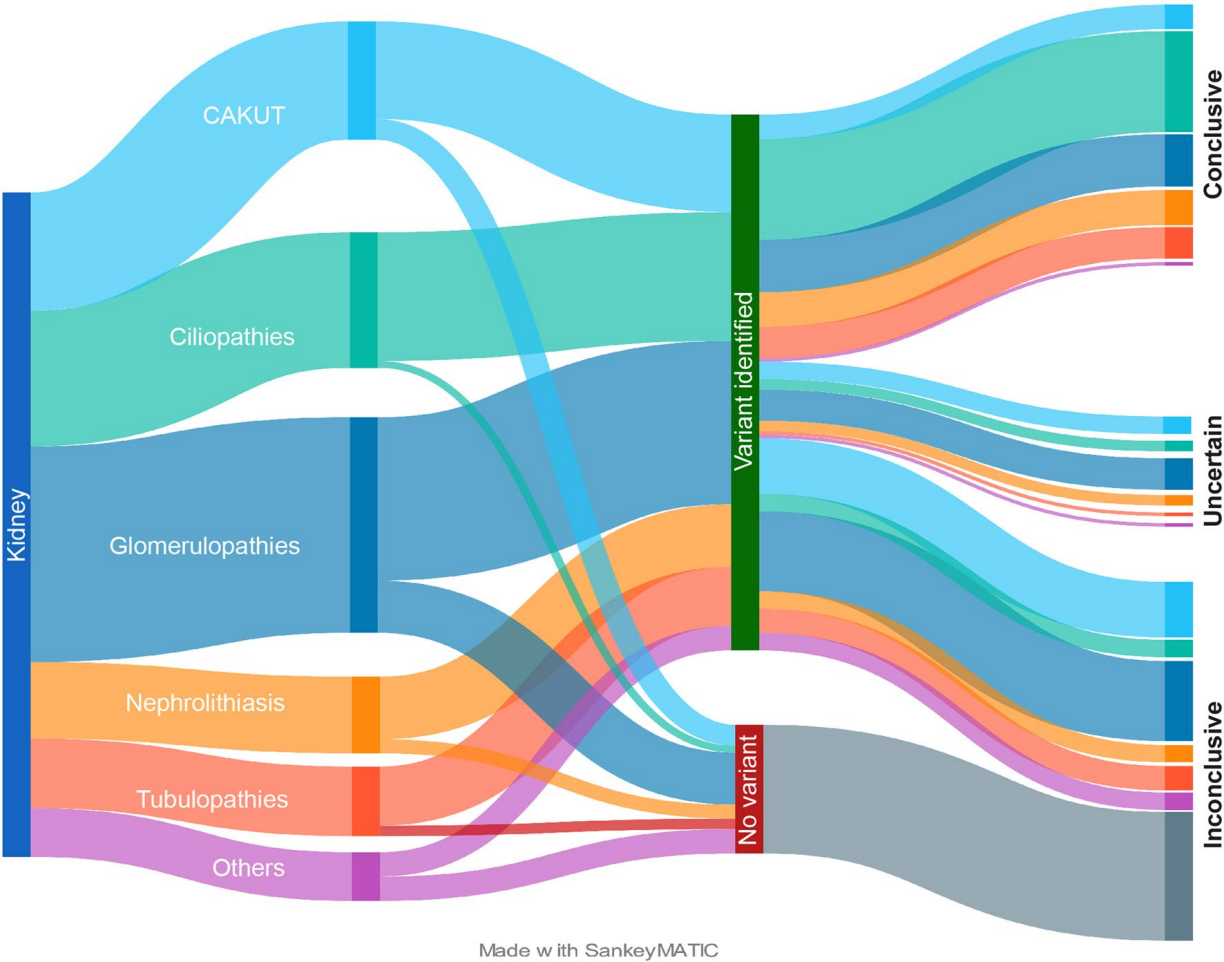
Representation of the cohort and workflow leading to the identification of causative variants. Sankey diagram summarizing the distribution of the cohort and the results of the analytical workflow. Nodes represent (i) the main disease macro-categories, (ii) variants identification or not by NGS, and (iii) classification of the genetic reports. Arrows width is proportional to the number of patients. The Sankey Matic tool was used to obtain the Sankey diagram

All patients underwent CES and were analyzed for variant prioritization and annotation following the criteria described above (Fig. 1). Overall, variants were detected in 154 patients (80.6%) with 37 patients (19.4%) presenting no variants. Sanger validation of the identified variant(s) and family segregation studies have been performed so far in 90 out of the 154 patients (58.4%). This approach allowed to (i) confirm the variants identified by CES in all cases, (ii) confirm their segregation with the phenotype, and (iii) identify de novo variants. Among the group of patients in which variants were identified by CES, a conclusive genetic report was obtained in 71 (46.1%; in 49 patients, variants were validated by Sanger sequencing and family segregation studies), while 22 (14.3%) and 61 (39.6%) patients remained with an uncertain genetic diagnosis or were classified as inconclusive, respectively (Fig. 3).

Overall, the application of CES followed by, whenever possible, family segregation studies allowed to reach a genetic diagnosis in a significant proportion of cases. The diagnostic yield is heterogenous when considering the different disease macro-categories with ciliopathies showing the higher diagnostic rate (74.4% of patients diagnosed), followed by nephrolithiasis and tubulopathies (45.5% and 45%, respectively), glomerulopathies and CAKUT (24.2 and 20.6%). The macro-category “others” that was the most heterogenous one presented the lowest diagnostic yield, with only 1 patient having a genetic diagnosis out of 14 (7.1%; Table 1). Not surprisingly, when considering a positive versus a negative family history, the former group of patients showed a higher diagnostic rate with 59.5% of patients diagnosed (Table 1).

**Table 1:** Diagnostic yield of clinical exome sequencing in pediatric cohort

Features	No of cases	No of cases with diagnostic variants	Diagnostic yield (%)
CAKUT	34	7	20.6
Ciliopathies	39	29	74.4
Glomerulopathies	62	15	24.2
Nephrolithiasis	22	10	45.5
Tubulopathies	20	9	45.0
Others	14	1	7.1
Positive family history	47	28	59.6
Negative family history	144	43	29.9

### Variant distribution and characteristics in the diagnosed cohort of patients

**Fig. 4:**
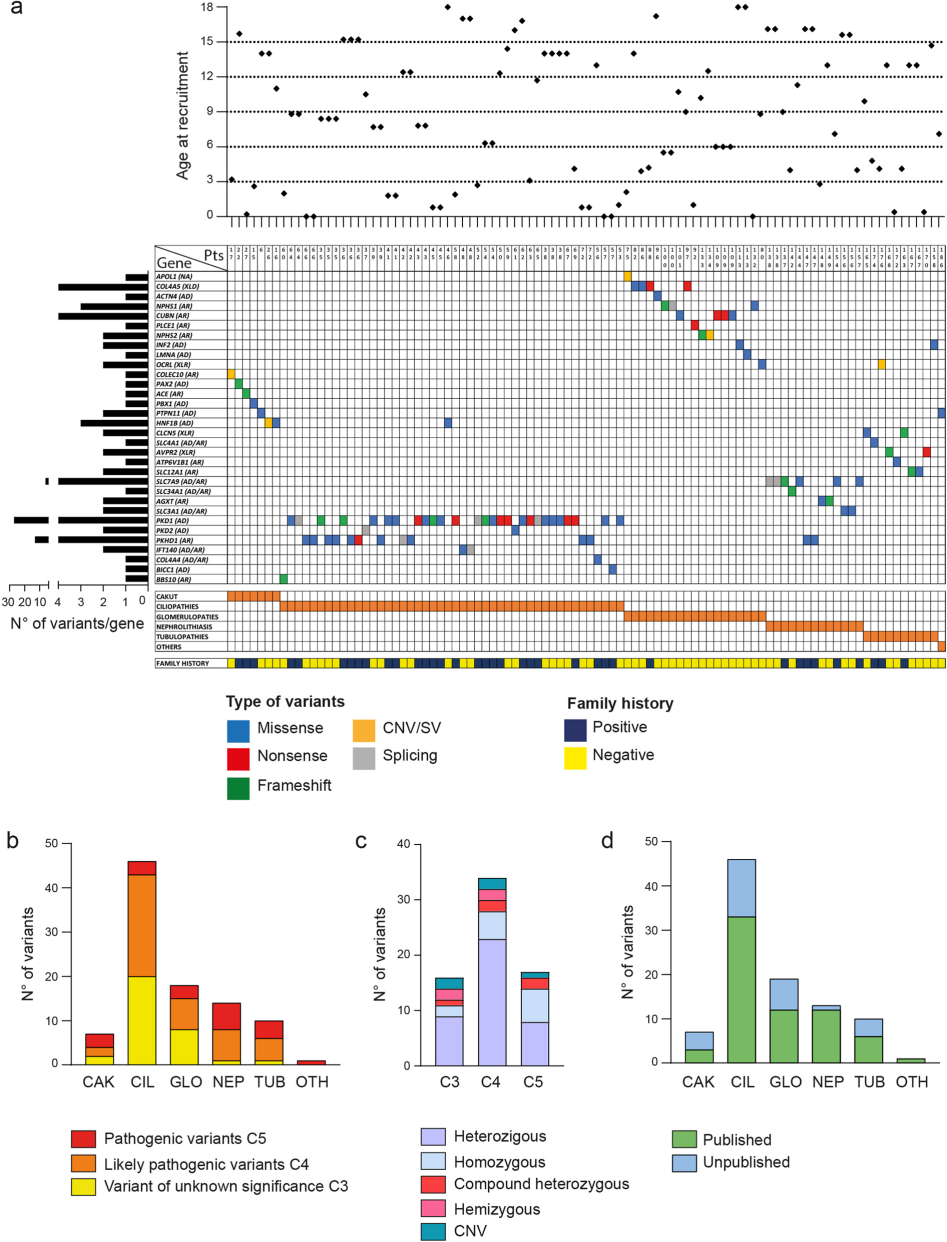
Summary of curated variants in disease-causative genes. The curated and reported variants are listed showing the gene involved and the type of variant (colored squares). Each raw represents a gene (mode of inheritance is reported in bracket; XLD: X-linked dominant; AD: autosomal dominant; AR: autosomal recessive; XLR: X-linked recessive; NA: not available) and each column a patient (Pts). Some patients presented more than one variant. Disease macro-categories (orange square) and family history (blue square for positive and yellow square for negative) for each patient are indicated. The graph of the top showed the age at recruitment for each diagnosed patient, while the histogram plot on the left showed the number of variants mapping within each gene (**a**). Number of variants and their ACMG classification identified for each disease macro-category (**b**). Distribution of heterozygous, homozygous, compound heterozygous, hemizygous and copy number variant (CNV) within the three different categories (C3-C4-C5) of variants (**c**). Histogram plot showing the distribution of the identified variants according to whether they are already or not published (**d**). CAK: CAKUT; CIL: ciliopathies; GLO: glomerulopathies; NEP: nephrolithiasis; TUB: tubulopathies; OTH: Others

Looking at the patients with a conclusive genetic report, 96 variants in 32 genes were identified and listed in patients’ genetic report (Fig. 4a and Additional file 1: Tables S1–S6). A few considerations can be drawn analyzing the data: (i) several patients presented with more than one variant either in the same or in different genes, not considering the compound heterozygous variants in recessive genes (e.g., #41, #43, #109; Fig. 4a and Additional file 1: Tables S2, S3). A significant proportion of these cases belong to the ciliopathies macro-category and specifically to polycystic kidney disease, posing the question whether additional variants within *PKD1* gene may have a clinical impact, leading to an earlier diagnosis (manuscript in preparation). (ii) The most recurrently mutated genes within the cohort were *COL4A5* and *PKD1*, in keeping with Alport and autosomal-dominant polycystic kidney disease (ADPKD) disease frequencies. (iii) Most of the identified variants are missense mutations (*n* = 53; 55.2%), followed by frameshift (*n* = 15; 15.6%), nonsense (n = 14; 145.6%) and splicing variants (*n* = 9; 9.4%). Moreover, 5 different CNVs were reported involving genes causative of CAKUT or glomerular diseases (5.2%; Fig. 4a and Additional file 1: Tables S1–S6). (iv) Nephrolithiasis and tubulopathies presented the highest number of C5 variants (*n* = 6 and *n* = 4, respectively) and the lowest number of C3 mutations (*n* = 1 each), which were on the contrary highly represented in ciliopathies (*n* = 20). C4 variants represented most mutations among all disease macro-categories (Fig. 4b).

(v) Within the conclusive genetic reports, CES resulted in the identification of pathogenic C5 variants in 17 patients (24%), likely pathogenic C4 variants in 34 (47.9%), variants of unknown significance-VUS C3 in 16 subjects (22.5%) and in 4 cases variants classified as C3/C4 (5.6%). When looking at the type of variants/frequency within patients and mode of inheritance, the majority of the C5 variants were heterozygous mutations (*n* = 8), followed by homozygous (*n* = 6) and compound heterozygous (*n* = 2) variants. A C5 CNV was part of this category. A similar pattern of distribution appeared for C4 and C3 variants, with heterozygous mutations being the most represented (*n* = 23 and *n* = 9, respectively; Fig. 4c).

(vi) No significant differences in the diagnostic rate were highlighted when considering European versus non-European subjects, even though it has to be taken in mind that the former group represented the great majority of the cohort.

Finally, (vii) independently of the disease category considered, most of the identified variants were already published and associated to specific clinical phenotype (Fig. 4d). Ciliopathies, and in particular polycystic kidney disease, was the only suspicion presenting a significant number of unpublished variants (13 out of 46), probably because of the higher number of variants identified compared to the other disease categories. All the identified variants are detailed in Additional file 1: Tables S1–S6.

## Discussion

Though rare in children, CKD has a profoundly negative impact on normal growth and development, compromising quantity and quality of life. The most recent analyses on adult and pediatric patients, who have received a kidney transplant or are included in the transplant waiting list or are present in the registries of the European Renal Association-European Dialysis and Transplant Association (ERA-EDTA) indicate that up to 27% of them are undiagnosed at the time of transplantation [7]. In line with these data, by analyzing the Transplant Registry of the Italian National Transplant Center, we recently reported that approximately 17.2% of the pediatric cohort was without a clear clinical diagnosis [6]. In addition, when considering the different disease categories, the great majority was affected by rare conditions and up to 50% by a monogenic disease [6]. These results suggest that genetic screening may be a valuable addition for increasing the diagnostic rate. It also represents a potent tool to confirm clinical diagnosis, as well as understanding the genetics underlining more complex or syndromic diseases, finally impacting on prognosis, management, and patients’ treatment.

Here, we report the results of the systematic use of a powerful genetic test, such CES in the diagnostic workflow of pediatric patients affected by nephropathies. Overall, a conclusive genetic test, based on CES followed by Sanger-based segregation studies, was obtained in 37.1% of patients, with a certain degree of heterogeneity when considering the different disease macro-categories. As expected, based on clinical presentation, the highest detection rates were obtained for ciliopathies (74.4%), followed by nephrolithiasis (45.5%) and tubular diseases (45%), while most glomerular diseases and CAKUT remained undiagnosed. In the case of glomerular diseases, a negative genetic test is important per se in that it rules out a structural cause for the disease, with significant implications for clinical management and transplantation outcome.

These data are in line with previously published results, even though some differences may be registered based on the group of patients considered, especially for highly homogeneous cohorts mainly based on same ethnic group (Table 2). It must be noted that many of the families where a VUS was identified are currently under investigation for variant segregation, most likely improving these performances.

**Table 2:** Comparison of the diagnostic yield

Disease macro-categories	Diagnostic yield					
CAKUT	20.6^§^	13.8 [15]	17 [16]	14 [17]	21.2 [18]	
Ciliopathies	74.4^§^	23.9 [19]	78 [20]	47.7 [21]		
Glomerulopathies	24.2^§^	7.2 [19]	62 [20]			
Nephrolithiasis	45.5^§^	14.9 [22]	16.8 [23]	29.4 [24]	33 [25]	75 [26]
Tubulopathies	45^§^	64 [27]	61.1 [21]			
Others	7.1^§^	28.6 [21]				

Comparison of the diagnostic yield between the present study and other published data of genetic analyses in pediatric cohorts

^§^Present study

These results underline the importance of an NGS-based genetic test together with family segregation studies and/or complementary tests (e.g., MLPA, array-CGH) as part of the routine diagnostic workflow. In addition to the relevance of having a diagnosis, these tests allow to identify other family member that may carry the same pathogenic variants as well as estimate the risk of disease recurrence. Moreover, considering that a significant percentage of these patients require kidney transplantation at some point, the availability of a genetic test to screen family member carries important implications in the selection of a live donor within the family.

A second point to be discussed is the importance to distinguish between genetic and non-genetic causes for some diseases. As an example, in the presence of a child with steroid resistant nephrotic syndrome (SRNS) it is essential to rule out conditions caused by mutations in genes coding for structural proteins of the podocyte. This can help to refine therapy, as children carrying pathogenic variants in podocyte genes generally do not benefit from immunosuppressive therapy or predicting prognosis, as “immunologic” SRNS is more likely to recur after transplantation. Genetic diagnosis may also result in fewer kidney biopsies, particularly for patients with glomerulopathy.

A third relevant consideration in favor of genetic testing in the clinical diagnostic workflow of pediatric patients is the translational impact of the identification of genetic variants. Indeed, there are actionable genes meaning that the corresponding disease conditions can be treated based on the presence of pathogenic variants, as in the case of renin-angiotensin blockade for patients carrying pathogenic variants in *COL4A3/COL4A4/COL4A5* genes. On the same line, having a genetic report may avoid useless or even deleterious treatment, such as immunosuppressive therapies for patients carrying mutations in collagen-coding genes [5]. Moreover, it can be useful for patients’ stratification and to assess potential risk of recurrence after a kidney transplant. As an example, patients diagnosed with atypical Hemolytic Uremic Syndrome and with a positive genetic report identifying pathogenic variants in *CFH*, *C3* or *CFB* genes, are at moderate to high risk of recurrence after transplantation [28]. For them, administration of eculizumab showed significant positive results with no relapse or relapse in a minority of cases, while its administration can be avoided for those patients at low risk [28–30].

A fourth point concerns family planning, as we are dealing with a pediatric population with parents that may wish to have additional children. The availability of a genetic diagnosis may be extremely useful for genetic counseling proposing to the couple all the available options for a future pregnancy. In line with this point, considering the present cohort, in 2 different cases a prenatal diagnosis was performed via Sanger sequencing, screening the fetus for the specific variant originally found by CES in the proband.

A fifth point that needs to be stressed concerns the number of C3-VUS variants identified by NGS, always posing a serious dilemma about their role in disease onset and progression and how they can be communicated during genetic counselling. In the last years, this topic has been addressed by setting up and designing novel computational methodologies that take in consideration not only nucleotide conservation, protein impact but also gene-association networks and pathway connections. Additional hints in deciphering the real meaning of C3 variants may come from transcriptomic analyses through the detection of aberrant expression or aberrant splicing mechanisms, as well as functional validation studies [31, 32]. Lastly, it is important to stress the relevance of periodic re-analysis of negative or inconclusive genetic reports and periodic re-evaluation of C3 variants. This kind of approach relies on (i) the discovery of new gene-disease/variant-disease associations, (ii) updated information from publicly available databases, (iii) re-classification of genetic variants based on functional evidence, (iv) amelioration of the in silico tools used for data alignment and variants annotation [33]. Up to now, no clear indication of the time interval after which negative/inconclusive cases or C3 variants must be re-analyzed has been provided by the Italian Society of Human Genetics (SIGU) or by the American College of Medical Genetics and Genomics guidelines. However, both these institutions suggest reviewing negative cases or variant classification either based on new findings by the lab or by external sources (e.g., literature or databases) or following clinicians’ request [34–36]. In line with this final point of discussion, it is worthy to note that in some cases, a single variant in recessive genes highly compatible with the clinical phenotype was found. While per se, these variants cannot explain clinical presentation, it is important to re-analyze and possibly to re-align original sequencing data to determine whether a second causative variant can be found.

A final point to be discussed is the financial impact of these tests for the National Healthcare system. In the Italian system, CES followed by analyses of a limited number of genes (< 8) is in the range of 1200 euros, all included, while larger panels are approximately double the amount. While these costs may seem elevated, a timely diagnosis may avoid unnecessary additional tests, including biopsies and may lead to optimized patient care and to early identification of family members with the same disease or at risk of developing the same disease. Family segregation studies are in the range of 150 euro per variant tested per person. In our view, to make this diagnostic workflow efficient and sustainable, it is necessary to identify local/regional “reference hubs” that can centralize these analyses, reducing costs and accumulating essential experience in variant calling and interpretation.

Overall, these results confirm the relevance of including routine genetic testing and counselling in the diagnostic workflow of pediatric patients affected by nephropathies where a monogenic condition is suspected or with a positive family history. For these patients genetic testing should be considered at the beginning of their diagnostic journey, as it may improve clinical management, spare unnecessary treatments, or diagnostic procedures, identify other family members potentially at risk of having the same genetic variants and, in case of kidney transplant, lead to optimal live-donor selection.

## Supplementary Information

**Additional file 1**. Tables reporting the diagnostic variants identified by clinical exome sequencing in CAKUT, ciliopathies, glomerulopathies, nephrolithiasis, tubulopathies and other kidney diseases.

## Abbreviations

NGS: Next-generation sequencing
CKD: Chronic kidney disease
ESRD: End-stage renal disease
IKD: Inherited kidney disease
CNV: Copy number variants
ACMG: American College of Medical Genetics and Genomics
MLPA: Multiplex ligation-dependent probe amplification
CES: Clinical exome sequencing
VUS: Variants of unknown significance
CAKUT: Congenital abnormalities of kidney and urinary tract
FSGS: Focal segmental glomerulosclerosis
