# Supplementary material for: The role of genetic testing in the diagnostic workflow of pediatric patients with kidney diseases: the experience of a single institution
Source: Hum Genomics. 2023 Feb 13;17:10. doi: 10.1186/s40246-023-00456-w (PMC9926680; doi:10.1186/s40246-023-00456-w)
Supplement: Supplementary file 1 — Additional file 1. Tables reporting the diagnostic variants identified by clinical exome sequencing in CAKUT, ciliopathies, glomerulopathies, nephrolithiasis, tubulopathies and other kidney diseases. [file 40246_2023_456_MOESM1_ESM.docx]

**Additional file 1**

| Pt ID | Gene | Chr | Chr location | Ref Seq | Exon/  Int | Variant | Type of variant | Amino acid change | Variant frq | ACMG clf | ClinVar clf | Varsome clf | Publication (0: no;  1: yes) |
| --- | --- | --- | --- | --- | --- | --- | --- | --- | --- | --- | --- | --- | --- |
| 17 | *COLEC10* | 8q24.12 | whole gene | NM_006438.5 | NA | Copy number loss | CNV | p.? | HOM | C5 | NA | C5 | 0 |
| 22 | *PAX2* | 10q24.31 | 102566320 | NM_003990.3 | 7 | c.821delC | Frameshift Indel | p.(Pro274Leufs*23) | HET | C4 | NA | C5 | 1 [1] |
| 27 | *ACE* | 17q23.3 | 61568738 | NM_000789.3 | 19 | c.2908_2909delTT | Frameshift Indel | p.(Phe970Profs*46) | HOM | C4 | NA | C5 | 0 |
| 15 | *PBX1* | 1q23 | 164776789 | NM_002585.3 | 5 | c.712C>T | Missense | p.(Arg238Trp) | HET | C3 | NA | NA | 0 |
| 6 | *PTPN11* | 12q24.13 | 112910827 | NM_002834.4 | 7 | c.836A>G | Missense | p.(Tyr279Cys) | HET | C5 | C5 | C5 | 1 [2] |
| 26 | *HNF1B* | 17q12 | whole gene | NM_000458.2 | NA | Copy number loss | CNV | p.? | HOM | C5 | NA | NA | 0 |
| 16 | *HNF1B* | 17q12 | 36099538 | NM_000458.2 | 2 | c.437A>T | Missense | p.(Asn146Ile) | HET | C3 | NA | C4 | 0 |
|  | | | | | | | | | | | | | |
| Table S1. Diagnostic variants identified by clinical exome sequencing in pediatric patients with a clinical suspicion of CAKUT. Pt: patient; Chr: chromosome; Ref Seq: reference sequence; Int: intron; CNV: copy number variant; frq: frequency; HOM: homozygous; HET: heterozygous; clf: classification. | | | | | | | | | | | | | |

| **Pt ID** | **Gene** | **Chr** | **Chr location** | **Ref Seq** | **Exon/**  **Int** | **Variant** | **Type of variant** | **Amino acid change** | **Variant frq** | **ACMG clf** | **ClinVar clf** | **Varsome clf** | **Publication**  **(0: no;**  **1: yes)** |
| --- | --- | --- | --- | --- | --- | --- | --- | --- | --- | --- | --- | --- | --- |
| 60 | *BBS10* | 12q21.2 | 76741493 | NM_024685.3 | 2 | c.271dupT | Frameshift Indel | p.(Cys91Leufs*5) | HOM | C5 | C5 | C5 | 1 [3] |
| 64 | *PKD1* | 16p13.3 | 2153765 | NM_001009944.2 | 23 | c.8293C>T | Missense | p.(Arg2765Cys) | HET | C4 | CIoP | C2 | 1 [4] |
|  | *PKD1* |  | 2158868 |  | 15 | c.6300G>A | Synonymous/  Splice region | p.(Ser2100Ser) | HET | C3 | NA | C2 | 0 |
| 66 | *PKHD1* | 6p12.3-p12.2 | 51619617 | NM_138694.3 | 4 | c.274C>G | Missense | p.(Arg92Gly) | HET | C4 | NA | C4 | 1 [5] |
|  | *PKHD1* |  | 51947197 |  | 56 | c.8762T>G | Missense | p.(Val2921Gly) | HET | C3 | NA | C3 | 1 [6] |
| 35 | *PKD1* | 16p13.3 | 2163229 | NM_001009944.2 | 12 | c.2917_2918delCA | Frameshift Indel | p.(Gln973Valfs*127) | HET | C4 | NA | C5 | 0 |
|  | *PKHD1* | 6p12.3-p12.2 | 51612709 | NM_138694.3 | 58 | c.652G>A | Missense | p.(Glu218Lys) | HET | C4 | CIoP | C4 | 1 [6] |
|  | *PKHD1* |  | 51935819 |  | 9 | c.9705T>A | Missense | p.(Asn3235Lys) | HET | C3 | C3 | C3 | 1 [7] |
| 36 | *PKD1* | 16p13.3 | 2141085 | NM_001009944.2 | 43 | c.11803delG | Frameshift Indel | p.(Ala3935Profs*10) | HET | C4 | NA | C4 | 1 [8] |
|  | *PKHD1* | 6p12.3-p12.2 | 51890158 | NM_138694.3 | 32 | c.4450G>A | Missense | p.(Ala1484Thr) | HET | C3 | CIoP | C3 | 1 [1] |
|  | *PKHD1* |  | 51484077 |  | 67 | c.12027C>G | Nonsense | p.(Tyr4009*) | HET | C3 | CIoP | C5 | 1 [9] |
| 37 | *PKD2* | 4q22.1 | 88968023 | NM_000297.3 | Int. 6 | c.1548+1G>A | Splicing | p.? | HET | C4 | C4 | C5 | 1 [10] |
| 39 | *PKD1* | 16p13.3 | 2153747 | NM_001009944.2 | 23 | c.8311G>A | Missense | p.(Glu2771Lys) | HET | C5 | C4/C5 | C5 | 1 [11] |
|  | *PKHD1* | 6p12.3-p12.2 | 51732719 | NM_138694.3 | 48 | c.7675G>C | Missense | p.(Val2559Leu) | HET | C3 | C3 | C3 | 1 [12] |
| 41 | *PKD1* | 16p13.3 | 2156545 | NM_001009944.2 | 18 | c.7343T>C | Missense | p.(Leu2448Pro) | HET | C3 | NA | C4 | 0 |
|  | *PKD1* |  | 2161396 |  | 15 | c.3772G>A | Missense | p.(Ala1258Thr) | HET | C3 | NA | C3 | 1 [13] |
| 42 | *PKHD1* | 6p12.3-p12.2 | 51947341 | NM_138694.3 | Int. 3 | c.131-1G>A | Splicing | p.? | HET | C4 | NA | C5 | 1 [1] |
|  | *PKHD1* |  | 51771090 |  | 41 | c.6731T>A | Missense | p.(Leu2244His) | HET | C3 | NA | C3 | 1 [6] |
| 43 | *PKD1* | 16p13.3 | 2156600 | NM_001009944.2 | 18 | c.7288C>T | Nonsense | p.(Arg2430*) | HET | C5 | C5 | C5 | 1 [14] |
|  | *PKD1* |  | 2150511 |  | 27 | c.9454C>T | Missense | p.(Arg3152Trp) | HET | C3 | C3 | C4 | 1 [15] |
| 45 | *PKD1* | 16p13.3 | 2159204 | NM_001009944.2 | 15 | c.5963_5964insG | Frameshift Indel | p.(Glu1989*) | HET | C4 | NA | C4 | 1 [1] |
|  | *PKD1* |  | 2156567 |  | 18 | c.7321G>A | Missense | p.(Gly2441Ser) | HET | C3 | C3 | C3 | 1 [15] |
| 46 | *HNF1B* | 17q12 | 36091725 | NM_000458.2 | 4 | c.906C>G | Missense | p.(Asn302Lys) | HET | C4 | NA | C4 | 1 [16] |
| 58 | *PKD1* | 16p13.3 | 2140406 | NM_001009944.2 | 45 | c.12324G>A | Nonsense | p.(Trp4108*) | HET | C4 | NA | C5 | 1 [1] |
| 48 | *IFT140* | 16p13.3 | 1576648 | NM_014714.3 | 20 | c.2549C>T | Missense | p.(Ala850Val) | HET | C3 | NA | C3 | 0 |
|  | *IFT140* |  | 1642470 |  | 5 | c.489C>T | Synonymous/  Splice region | p.(Gly163=) | HET | C3 | C3 | C4 | 0 |
| 52 | *PKD1* | 16p13.3 | 2162339 | NM_001009944.2 | Int. 14 | c.3295+2T>C | Splicing | p.? | HET | C4 | NA | C5 | 1 [13] |
| 54 | *PKD1* | 16p13.3 | 2159190 | NM_001009944.2 | 15 | c.5976_5978delCAC | Inframe indel | p.(Phe1992_  Thr1993delinsLeu) | HET | C4 | C4 | C4 | 1 [14] |
|  | *PKD1* |  | 2160409 |  | 15 | c.4759C>T | Missense | p.(Arg1587Cys) | HET | C3 | NA | C3 | 1 [13] |
| 50 | *PKD1* | 16p13.3 | 2140406 | NM_001009944.2 | 45 | c.12324G>A | Nonsense | p.(Trp4108*) | HET | C4 | NA | C5 | 1 [1] |
| 59 | *PKD1* | 16p13.3 | 2160579 | NM_001009944.2 | 15 | c.4589G>A | Nonsense | p.(Trp1530*) | HET | C4 | NA | C5 | 0 |
| 61 | *PKD2* | 4q22.1 | 88977278 | NM_000297.3 | 8 | c.1757T>C | Missense | p.(Leu586Pro) | HET | C3 | NA | C3 | 0 |
| 62 | *PKD1* | 16p13.3 | 2153303 | NM_001009944.2 | 23 | c.8755G>A | Missense | p.(Gly2919Arg) | HET | C4 | C3 | C3 | 0 |
| 63 | *PKD1* | 16p13.3 | 2158297 | NM_001009944.2 | 15 | c.6871C>T | Nonsense | p.(Gln2291Ter) | HET | C4 | C5 | C5 | 0 |
| 65 | *PKD1* | 16p13.3 | 2169115 | NM_001009944.2 | 3 | c.359T>C | Splicing | p.(Ile120Thr) | HET | C4 | C4 | C4 | 1 [8] |
| 38 | *PKD1* | 16p13.3 | 2140192 | NM_001009944.2 | 46 | c.12448C>T | Missense | p.(Arg4150Cys) | HET | C4 | C4/C5 | C5 | 1 [8] |
|  | *PKD1* |  | 2140180 |  | 46 | c.12460C>T | Missense | p.(Arg4154Cys) | HET | C4 | CIoP | C4 | 1 [17] |
|  | *PKD1* |  | 2143597 |  | 37 | c.10964T>C | Missense | p.(Phe3655Ser) | HET | C3 | NA | C3/C4 | 0 |
| 67 | *PKD1* | 16p13.3 | 2139949 | NM_001009944.2 | 46 | c.12691C>T | Nonsense | p.(Gln4231*) | HET | C4 | C4/C5 | C5 | 1 [14] |
| 69 | *PKD1* | 16p13.3 | 2158744 | NM_001009944.2 | 15 | c.6424C>T | Nonsense | p.(Gln2142*) | HET | C4 | NA | C5 | 1 [13] |
| 72 | *PKHD1* | 6p12.3-p12.2 | 51889726 | NM_138694.3 | 32 | c.4882C>G | Missense | p.(Pro1628Ala) | HET | C4 | C4/C5 | C4 | 1 [6] |
|  | *PKHD1* |  | 51612950 |  | 58 | c.9464A>G | Missense | p.(Tyr3155Cys) | HET | C3 | NA | C3 | 1 [5] |
| 56 | *COL4A4* | 2q36.3 | 227968767 | NM_000092.4 | 13 | c.737G>T | Missense/  splice region | p.(Gly246Val) | HET | C4 | NA | C5 | 0 |
| 57 | *BICC1* | 10q21.1 | 60562958 | NM_001080512.2 | 15 | c.2137G>A | Missense | p.(Glu713Lys) | HET | C3 | NA | C3 | 0 |
|  | *PKD1* | 16p13.3 | 2140180 | NM_001009944.2 | 46 | c.12460C>T | Missense | p.(Arg4154Cys) | HET | C3 | C3 | C4 | 1 [17] |
| 53 | *PKD1* | 16p13.3 | 2156617 | NM_001009944.2 | 18 | c.7271C>T | Missense | p.(Thr2424Met) | HET | C3 | NA | C4 | 0 |
|  | | | | | | | | | | | | | |
| **Table S2. Diagnostic variants identified by clinical exome sequencing in pediatric patients with a clinical suspicion of ciliopathies.** Pt: patient; Chr: chromosome; Ref Seq: reference sequence; Int: intron; CNV: copy number variant; frq: frequency; HOM: homozygous; HET: heterozygous; CIoP: conflicted interpretation of pathogenicity; clf: classification. | | | | | | | | | | | | | |

| Pt ID | Gene | Chr | Chr location | Ref Seq | Exon/  Int | Variant | Type of variant | Amino acid change | Variant frq | ACMG clf | ClinVar clf | Varsome clf | Publication (0: no;  1: yes) |
| --- | --- | --- | --- | --- | --- | --- | --- | --- | --- | --- | --- | --- | --- |
| 75 | *APOL1* | 22q12.3 | 36597688-36651398 | NA | 1-2 | Inversion | SV | NA | NA | C3 | NA | NA | 1 [18] |
| 82 | *COL4A5* | Xq22.3 | 107911648 | NM_033380.2 | 41 | c.3704G>A | Missense | p.(Gly1235Asp) | HOM | C4 | NA | C5 | 0 |
| 86 | *COL4A5* | Xq22.3 | 107846275 | NM_033380.2 | 28 | c.2228G>T | Missense | p.(Gly743Val) | HET | C4 | NA | C4 | 1 [19] |
| 88 | *COL4A5* | Xq22.3 | 107936078 | NM_033380.2 | 50 | c.4629C>A | Nonsense | p.(Tyr1543*) | HEM | C4 | NA | C5 | 1 [1] |
| 96 | *ACTN4* | 19q13.2 | 39195635 | NM_004924.5 | 4 | c.459C>G | Missense | p.(Phe153Leu) | HET | C3 | NA | C4 | 1 [1] |
| 100 | *NPHS1* | 19q13.12 | 36330397 | NM_004646.3 | Int. 21 | c.2927+1G>A | Splicing | p.? | HET | C4 | C4 | C5 | 1 [20] |
|  | *NPHS1* |  | 36333134 |  | 19 | c.2542_2555del  AAGGTGGCTGCAGC  insCAGG | Frameshift indel | p.(Lys848GlnfsTer54) | HET | C4 | NA | C5 | 0 |
| 101 | *CUBN* | 10p13 | 17171208 | NM_001081.3 | 2 | c.164C>T | Missense | p.(Thr55Met) | HOM | C3 | C3 | C3 | 1 [1] |
| 97 | *COL4A5* | Xq22.3 | 107936130 | NM_033380.2 | 50 | c.4681C>T | Nonsense | p.(Gln1561*) | HET | C4 | NA | C5 | 0 |
| 92 | *PLCE1* | 10q23.33 | 95791764 | NM_016341.3 | 2 | c.961C>T | Nonsense | p.(Arg321*) | HOM | C5 | C5 | C5 | 1 [21] |
| 133 | *NPHS2* | 1q25.2 | 179521755 | NM_014625.2 | 7 | c.855_856delAA | Frameshift indel | p.(Arg286Thrfs*17) | HOM | C4 | NA | C5 | 1 [22] |
| 134 | *NPHS2* | 1q25.2 | 179520297-179528906 | NA | 2-8 | Copy number gain | CNV | NA | NA | C3 | NA | NA | 0 |
| 109 | *CUBN* | 10p13 | 16948208 | NM_001081.3 | 50 | c.7906C>T | Nonsense | p.(Arg2636*) | HET | C5 | C5 | C5 | 1 [23] |
|  | *CUBN* |  | 17024488-17024489 |  | 31 | c.4689_4690delTA  insAT | Nonsense | p.(Cys1563_Ile1564  delins*) | HET | C5 | C5 | C5 | 1 [24] |
|  | *CUBN* |  | 16960659 |  | 45 | c.6962G>T | Missense | p.(Ser2321Ile) | HET | C3 | C3 | C3 | 0 |
| 113 | *INF2* | 14q32.33 | 105169678 | NM_022489.3 | 4 | c.554T>C | Missense | p.(Leu185Pro) | HET | C3 | C3 | C3 | 0 |
|  | *LMNA* | 1q22 | 156107470 | NM_170707.3 | 10 | c.1634G>A | Missense | p.(Arg545His) | HET | C3 | CIoP | C4 | 1 [25] |
| 132 | *NPHS1* | 19q13.12 | 36339004 | NM_004646.3 | 11 | c.1379G>A | Missense | p.(Arg460Gln) | HOM | C5 | C4/C5 | C5 | 1 [26] |
| 80 | *OCRL* | Xq26.1 | 128721050 | NM_001318784.1 | 20 | c.2214G>T | Missense | p.(Glu738Asp) | HEM | C3 | NA | C4 | 0 |
|  | | | | | | | | | | | | | |
| Table S3. Diagnostic variants identified by clinical exome sequencing in pediatric patients with a clinical suspicion of glomerulopathies. Pt: patient; Chr: chromosome; Ref Seq: reference sequence; Int: intron; SV: structural variant; CNV: copy number variant; frq: frequency; HOM: homozygous; HET: heterozygous; clf: classification. | | | | | | | | | | | | | |

| Pt ID | Gene | Chr | Chr location | Ref Seq | Exon/  Int | Variant | Type of variant | Amino acid change | Variant frq | ACMG clf | ClinVar clf | Varsome clf | Publication (0: no;  1: yes) |
| --- | --- | --- | --- | --- | --- | --- | --- | --- | --- | --- | --- | --- | --- |
| 138 | *SLC7A9* | 19q13.11 | 33353365 | NM_014270.4 | Int. 5 | c.604+2T>C | Splicing | p.? | HET | C5 | C3/C5 | C5 | 1 [27] |
|  | *SLC7A9* |  | 33324052 |  | Int. 12 | c.1399+2dupT | Splicing | p.? | HET | C3 | NA | C3 | 1 [28] |
| 137 | *SLC7A9* | 19q13.11 | 33333198 | NM_014270.4 | 11 | c.1099dupA | Frameshift Indel | p.(Ile367Asnfs*4) | HET | C4 | NA | C5 | 0 |
| 142 | *SLC34A1* | 5q35.3 | 176813233 | NM_003052.4 | 4 | c.272_292del TCCCCAAGCTGCGCCAGGCTG | Inframe deletion | p.(Val91_Ala97del) | HET | C4 | CIoP | C4 | 1 [29] |
| 144 | *SLC7A9* | 19q13.11 | 33355167 | NM_014270.4 | 4 | c.313G>A | Missense | p.(Gly105Arg) | HET | C5 | C5 | C5 | 1 [30] |
| 147 | *PKHD1* | 6p12.3-p12.2 | 51889738 | NM_138694.3 | 32 | c.4870C>T | Missense | p.(Arg1624Trp) | HET | C4 | C5 | C4 | 1 [31] |
|  | *PKHD1* |  | 51613044 |  | 58 | c.9370C>T | Missense | p.(His3124Tyr) | HET | C4 | C4/C5 | C4 | 1 [14] |
| 148 | *AGXT* | 2q37.3 | 241814576 | NM_000030.2 | 7 | c.731T>C | Missense | p.(Ile244Thr) | HOM | C5 | C5 | C5 | 1 [32] |
| 149 | *AGXT* | 2q37.3 | 241808307 | NM_000030.2 | 1 | c.33dupC | Frameshift Indel | p.(Lys12Glnfs*156) | HOM | C5 | NA | C5 | 1 [32] |
| 154 | *SLC7A9* | 19q13.11 | 33355167 | NM_014270.4 | 4 | c.313G>A | Missense | p.(Gly105Arg) | HET | C5 | C5 | C5 | 1 [30] |
| 156 | *SLC3A1* | 2p21 | 44539792 | NM_000341.3 | 8 | c.1400T>C | Missense | p.(Met467Thr) | HET | C5 | C5 | C5 | 1 [33] |
|  | *SLC3A1* |  | 44547360 |  | 10 | c.1640C>G | Missense | p.(Ser547Trp) | HET | C4 | C5 | C4 | 1 [33] |
| 157 | *SLC7A9* | 19q13.11 | 33353057 | NM_014270.4 | 6 | c.671C>T | Missense | p.(Ala224Val) | HET | C4 | C5 | C4 | 1 [27] |
|  | | | | | | | | | | | | | |
| Table S4. Diagnostic variants identified by clinical exome sequencing in pediatric patients with a clinical suspicion of nephrolithiasis. Pt: patient; Chr: chromosome; Ref Seq: reference sequence; Int: intron; CNV: copy number variant; frq: frequency; HOM: homozygous; HET: heterozygous; clf: classification. | | | | | | | | | | | | | |

| Pt ID | Gene | Chr | Chr location | Ref Seq | Exon/  Int | Variant | Type of variant | Amino acid change | Variant frq | ACMG clf | ClinVar clf | Varsome clf | Publication (0: no;  1: yes) |
| --- | --- | --- | --- | --- | --- | --- | --- | --- | --- | --- | --- | --- | --- |
| 165 | *CLCN5* | Xp11.23 | 49850710 | NM_001127898.2 | 10 | c.1007T>C | Missense | p.(Leu336Pro) | HEM | C4 | NA | C4 | 1 [1] |
| 174 | *SLC4A1* | 17q21.31 | 42333076 | NM_000342.3 | 14 | c.1765C>T | Missense | p.(Arg589Cys) | HET | C5 | C4/C5 | C5 | 1 [34] |
| 176 | *OCRL* | Xq25q26.1 | 128581128 -128724257 | NA | Whole gene | Deletion | CNV | p.? | HEM | C5 | NA | NA | 0 |
| 168 | *AVPR2* | Xq28 | 153171102 | NM_000054.4 | 2 | c.143_144delTT | Frameshift Indel | p.(Phe48Cysfs*143) | HEM | C5 | NA | C5 | 1 [35] |
| 172 | *ATP6V1B1* | 2p13.3 | 71185243 | NM_001692.3 | 3 | c.242T>C | Missense | p.(Leu81Pro) | HOM | C5 | C4/C5 | C4 | 1 [36] |
| 163 | *CLCN5* | Xp11.23 | 49854895 | NM_001127898.3 | 13 | c.1869delG | Frameshift Indel | p.(Arg624Glyfs*32) | HEM | C4 | NA | C5 | 1 [1] |
| 167 | *SLC12A1* | 15q21.1 | 48527094 | NM_000338.2 | 9 | c.1110delT | Frameshift Indel | p.(Phe370LeufsTer58) | HET | C4 | NA | C4 | 0 |
|  | *SLC12A1* |  | 48566791 |  | 20 | c.2426G>A | Missense | p.(Gly809Asp) | HET | C3 | NA | C3 | 0 |
| 170 | *AVPR2* | Xq28 | 153171847 | NM_000054.4 | 2 | c.887G>A | Nonsense | p.(Trp296*) | HEM | C4 | NA | C5 | 1 [37] |
| 158 | *INF2* | 14q32.33 | 105169520 | NM_022489.3 | 3 | c.470G>A | Missense | p.(Gly157Asp) | HET | C4 | C3 | C4 | 0 |
|  | | | | | | | | | | | | | |
| Table S5. Diagnostic variants identified by clinical exome sequencing in pediatric patients with a clinical suspicion of tubulopathies. Pt: patient; Chr: chromosome; Ref Seq: reference sequence; Int: intron; CNV: copy number variant; frq: frequency; HOM: homozygous; HET: heterozygous; clf: classification. | | | | | | | | | | | | | |

| Pt ID | Gene | Chr | Chr location | Ref Seq | Exon/  Int | Variant | Type of variant | Amino acid change | Variant frq | ACMG clf | ClinVar clf | Varsome clf | Publication (0: no; 1: yes) |
| --- | --- | --- | --- | --- | --- | --- | --- | --- | --- | --- | --- | --- | --- |
| 186 | *PTPN11* | 12q24.13 | 112926851 | NM_002834.3 | 13 | c.1471C>T | Missense | p.(Pro491Ser) | HET | C5 | C5 | C5 | 1 [38] |
|  | | | | | | | | | | | | | |
| Table S6. Diagnostic variants identified by clinical exome sequencing in pediatric patients with a clinical suspicion of other kidney diseases. Pt: patient; Chr: chromosome; Ref Seq: reference sequence; Int: intron; CNV: copy number variant; frq: frequency; HOM: homozygous; HET: heterozygous; clf: classification. | | | | | | | | | | | | | |

**References**

1. Vaisitti T, Sorbini M, Callegari M, Kalantari S, Bracciama V, Arruga F, Vanzino SB, Rendine S, Togliatto G, Giachino D *et al*: Clinical exome sequencing is a powerful tool in the diagnostic flow of monogenic kidney diseases: an Italian experience. *J Nephrol* 2021, 34(5):1767-1781.

2. Mutai H, Momozawa Y, Kamatani Y, Nakano A, Sakamoto H, Takiguchi T, Nara K, Kubo M, Matsunaga T: Whole exome analysis of patients in Japan with hearing loss reveals high heterogeneity among responsible and novel candidate genes. *Orphanet J Rare Dis* 2022, 17(1):114.

3. Stoetzel C, Laurier V, Davis EE, Muller J, Rix S, Badano JL, Leitch CC, Salem N, Chouery E, Corbani S *et al*: BBS10 encodes a vertebrate-specific chaperonin-like protein and is a major BBS locus. *Nat Genet* 2006, 38(5):521-524.

4. Nielsen ML, Lildballe DL, Rasmussen M, Bojesen A, Birn H, Sunde L: Clinical genetic diagnostics in Danish autosomal dominant polycystic kidney disease patients reveal possible founder variants. *Eur J Med Genet* 2021, 64(4):104183.

5. Bergmann C, Senderek J, Windelen E, Kupper F, Middeldorf I, Schneider F, Dornia C, Rudnik-Schoneborn S, Konrad M, Schmitt CP *et al*: Clinical consequences of PKHD1 mutations in 164 patients with autosomal-recessive polycystic kidney disease (ARPKD). *Kidney Int* 2005, 67(3):829-848.

6. Melchionda S, Palladino T, Castellana S, Giordano M, Benetti E, De Bonis P, Zelante L, Bisceglia L: Expanding the mutation spectrum in 130 probands with ARPKD: identification of 62 novel PKHD1 mutations by sanger sequencing and MLPA analysis. *J Hum Genet* 2016, 61(9):811-821.

7. Alehabib E, Jamshidi J, Ghaedi H, Askarian F, Mahmoudieh L, Johari AH, Darvish H: Bioinformatic tools to determine the pathogenicity of a missense mutation in PKHD1 in autosomal recessive polycystic kidney disease. *Nephrology (Carlton)* 2017, 22(4):330-331.

8. Audrezet MP, Cornec-Le Gall E, Chen JM, Redon S, Quere I, Creff J, Benech C, Maestri S, Le Meur Y, Ferec C: Autosomal dominant polycystic kidney disease: comprehensive mutation analysis of PKD1 and PKD2 in 700 unrelated patients. *Hum Mutat* 2012, 33(8):1239-1250.

9. Sallevelt S, Stegmann APA, de Koning B, Velter C, Steyls A, van Esch M, Lakeman P, Yntema H, Esteki MZ, de Die-Smulders CEM *et al*: Diagnostic exome-based preconception carrier testing in consanguineous couples: results from the first 100 couples in clinical practice. *Genet Med* 2021, 23(6):1125-1136.

10. Cornec-Le Gall E, Audrezet MP, Renaudineau E, Hourmant M, Charasse C, Michez E, Frouget T, Vigneau C, Dantal J, Siohan P *et al*: PKD2-Related Autosomal Dominant Polycystic Kidney Disease: Prevalence, Clinical Presentation, Mutation Spectrum, and Prognosis. *Am J Kidney Dis* 2017, 70(4):476-485.

11. Munch J, Kirschner KM, Schlee H, Kraus C, Schonauer R, Jin W, Le Duc D, Scholz H, Halbritter J: Autosomal dominant polycystic kidney disease in absence of renal cyst formation illustrates genetic interaction between WT1 and PKD1. *J Med Genet* 2020.

12. Tavira B, Gomez J, Malaga S, Santos F, Fernandez-Aracama J, Alonso B, Iglesias S, Benavides A, Hernando I, Plasencia A *et al*: A labor and cost effective next generation sequencing of PKHD1 in autosomal recessive polycystic kidney disease patients. *Gene* 2015, 561(1):165-169.

13. Carrera P, Calzavara S, Magistroni R, den Dunnen JT, Rigo F, Stenirri S, Testa F, Messa P, Cerutti R, Scolari F *et al*: Deciphering Variability of PKD1 and PKD2 in an Italian Cohort of 643 Patients with Autosomal Dominant Polycystic Kidney Disease (ADPKD). *Sci Rep* 2016, 6:30850.

14. Schonauer R, Baatz S, Nemitz-Kliemchen M, Frank V, Petzold F, Sewerin S, Popp B, Munch J, Neuber S, Bergmann C *et al*: Matching clinical and genetic diagnoses in autosomal dominant polycystic kidney disease reveals novel phenocopies and potential candidate genes. *Genet Med* 2020, 22(8):1374-1383.

15. Lanktree MB, Haghighi A, Guiard E, Iliuta IA, Song X, Harris PC, Paterson AD, Pei Y: Prevalence Estimates of Polycystic Kidney and Liver Disease by Population Sequencing. *J Am Soc Nephrol* 2018, 29(10):2593-2600.

16. Heidet L, Decramer S, Pawtowski A, Moriniere V, Bandin F, Knebelmann B, Lebre AS, Faguer S, Guigonis V, Antignac C *et al*: Spectrum of HNF1B mutations in a large cohort of patients who harbor renal diseases. *Clin J Am Soc Nephrol* 2010, 5(6):1079-1090.

17. Audrezet MP, Corbiere C, Lebbah S, Moriniere V, Broux F, Louillet F, Fischbach M, Zaloszyc A, Cloarec S, Merieau E *et al*: Comprehensive PKD1 and PKD2 Mutation Analysis in Prenatal Autosomal Dominant Polycystic Kidney Disease. *J Am Soc Nephrol* 2016, 27(3):722-729.

18. Ahn SM, Kim TH, Lee S, Kim D, Ghang H, Kim DS, Kim BC, Kim SY, Kim WY, Kim C *et al*: The first Korean genome sequence and analysis: full genome sequencing for a socio-ethnic group. *Genome Res* 2009, 19(9):1622-1629.

19. Marinakis NM, Svingou M, Veltra D, Kekou K, Sofocleous C, Tilemis FN, Kosma K, Tsoutsou E, Fryssira H, Traeger-Synodinos J: Phenotype-driven variant filtration strategy in exome sequencing toward a high diagnostic yield and identification of 85 novel variants in 400 patients with rare Mendelian disorders. *Am J Med Genet A* 2021, 185(8):2561-2571.

20. Schoeb DS, Chernin G, Heeringa SF, Matejas V, Held S, Vega-Warner V, Bockenhauer D, Vlangos CN, Moorani KN, Neuhaus TJ *et al*: Nineteen novel NPHS1 mutations in a worldwide cohort of patients with congenital nephrotic syndrome (CNS). *Nephrol Dial Transplant* 2010, 25(9):2970-2976.

21. Tokhmafshan F, Dickinson K, Akpa MM, Brasell E, Huertas P, Goodyer PR: A no-nonsense approach to hereditary kidney disease. *Pediatr Nephrol* 2020, 35(11):2031-2042.

22. Miko A, D KM, Kaposi A, Antignac C, Tory K: The mutation-dependent pathogenicity of NPHS2 p.R229Q: A guide for clinical assessment. *Hum Mutat* 2018, 39(12):1854-1860.

23. Mejecase C, Hummel A, Mohand-Said S, Andrieu C, El Shamieh S, Antonio A, Condroyer C, Boyard F, Foussard M, Blanchard S *et al*: Whole exome sequencing resolves complex phenotype and identifies CC2D2A mutations underlying non-syndromic rod-cone dystrophy. *Clin Genet* 2019, 95(2):329-333.

24. Domingo-Gallego A, Pybus M, Bullich G, Furlano M, Ejarque-Vila L, Lorente-Grandoso L, Ruiz P, Fraga G, Lopez Gonzalez M, Pinero-Fernandez JA *et al*: Clinical utility of genetic testing in early-onset kidney disease: seven genes are the main players. *Nephrol Dial Transplant* 2022, 37(4):687-696.

25. van Tienen FHJ, Lindsey PJ, Kamps MAF, Krapels IP, Ramaekers FCS, Brunner HG, van den Wijngaard A, Broers JLV: Assessment of fibroblast nuclear morphology aids interpretation of LMNA variants. *Eur J Hum Genet* 2019, 27(3):389-399.

26. Ohmori T, De S, Tanigawa S, Miike K, Islam M, Soga M, Era T, Shiona S, Nakanishi K, Nakazato H *et al*: Impaired NEPHRIN localization in kidney organoids derived from nephrotic patient iPS cells. *Sci Rep* 2021, 11(1):3982.

27. Tkaczyk M, Gadomska-Prokop K, Zaluska-Lesniewska I, Musial K, Zawadzki J, Jobs K, Porowski T, Rogowska-Kalisz A, Jander A, Kirolos M *et al*: Clinical profile of a Polish cohort of children and young adults with cystinuria. *Ren Fail* 2021, 43(1):62-70.

28. Li C, Yang Y, Zheng Y, Shen F, Liu L, Li Y, Li L, Zhao Y: Genetic and Clinical Analyses of 13 Chinese Families With Cystine Urolithiasis and Identification of 15 Novel Pathogenic Variants in SLC3A1 and SLC7A9. *Front Genet* 2020, 11:74.

29. Garcia-Castano A, Madariaga L, Gomez-Conde S, Cordo CLR, Lopez-Iglesias M, Garcia-Fernandez Y, Martin A, Gonzalez P, Goicolea I, de Nanclares GP *et al*: Five patients with disorders of calcium metabolism presented with GCM2 gene variants. *Sci Rep* 2021, 11(1):2968.

30. Zacchia M, Blanco FDV, Trepiccione F, Blasio G, Torella A, Melluso A, Capolongo G, Pollastro RM, Piluso G, Di Iorio V *et al*: Nephroplex: a kidney-focused NGS panel highlights the challenges of PKD1 sequencing and identifies a founder BBS4 mutation. *J Nephrol* 2021, 34(6):1855-1874.

31. Al-Hamed MH, Hussein MH, Shah Y, Al-Mojalli H, Alsabban E, Alshareef T, Altayyar A, Elshouny S, Ali W, Abduljabbar M *et al*: Exome sequencing unravels genetic variants associated with chronic kidney disease in Saudi Arabian patients. *Hum Mutat* 2022, 43(12):e24-e37.

32. Soliman NA, Elmonem MA, Abdelrahman SM, Nabhan MM, Fahmy YA, Cogal A, Harris PC, Milliner DS: Clinical and molecular characterization of primary hyperoxaluria in Egypt. *Sci Rep* 2022, 12(1):15886.

33. Botzenhart E, Vester U, Schmidt C, Hesse A, Halber M, Wagner C, Lang F, Hoyer P, Zerres K, Eggermann T *et al*: Cystinuria in children: distribution and frequencies of mutations in the SLC3A1 and SLC7A9 genes. *Kidney Int* 2002, 62(4):1136-1142.

34. Gomez-Conde S, Garcia-Castano A, Aguirre M, Herrero M, Gondra L, Garcia-Perez N, Garcia-Ledesma P, Martin-Penagos L, Dall'Anese C, Ariceta G *et al*: Molecular aspects and long-term outcome of patients with primary distal renal tubular acidosis. *Pediatr Nephrol* 2021, 36(10):3133-3142.

35. Hong CR, Kang HG, Choi HJ, Cho MH, Lee JW, Kang JH, Park HW, Koo JW, Ha TS, Kim SY *et al*: X-linked recessive nephrogenic diabetes insipidus: a clinico-genetic study. *J Pediatr Endocrinol Metab* 2014, 27(1-2):93-99.

36. Daga A, Majmundar AJ, Braun DA, Gee HY, Lawson JA, Shril S, Jobst-Schwan T, Vivante A, Schapiro D, Tan W *et al*: Whole exome sequencing frequently detects a monogenic cause in early onset nephrolithiasis and nephrocalcinosis. *Kidney Int* 2018, 93(1):204-213.

37. Daitsu T, Igaki J, Goto M, Hasegawa Y: A novel deletion mutation of the arginine vasopressin receptor 2 gene in a Japanese infant with nephrogenic diabetes insipidus. *Clin Pediatr Endocrinol* 2014, 23(4):115-117.

38. Baldo F, Fachin A, Da Re B, Rubinato E, Bobbo M, Barbi E: New insights on Noonan syndrome's clinical phenotype: a single center retrospective study. *BMC Pediatr* 2022, 22(1):734.
